# Prevalence and detection of *Stenotrophomonas maltophilia* carrying metallo-β-lactamase *bla*L1 in Beijing, China

**DOI:** 10.3389/fmicb.2014.00692

**Published:** 2014-12-09

**Authors:** Zhan Yang, Wei Liu, Qian Cui, Wenkai Niu, Huan Li, Xiangna Zhao, Xiao Wei, Xuesong Wang, Simo Huang, Derong Dong, Sijing Lu, Changqing Bai, Yan Li, Liuyu Huang, Jing Yuan

**Affiliations:** ^1^Institute of Disease Control and Prevention, Academy of Military Medical SciencesBeijing, China; ^2^Department of Respiratory Diseases, Affiliated Hospital of Academy of Military Medical SciencesBeijing, China; ^3^Department of Respiration, The First Affiliated Hospital of Liaoning Medical UniversityJinzhou, China

**Keywords:** L1 metallo-β-lactamase, *S. maltophilia*, LAMP, rapid diagnosis, prevalence

## Abstract

Intrinsic β-lactam resistance in *Stenotrophomonas maltophilia* is caused by *bla*_L1_ and/or *bla*_L2_, a kind of metallo-β-lactamase with a broad substrate spectrum including carbapenems. A rapid and sensitive molecular method for the detection of *bla*_L1_ in clinical samples is needed to guide therapeutic treatment. In present study, we first described a loop-mediated isothermal amplification (LAMP) method for the rapid detection of *bla*_L1_ in clinical samples by using two methods including a chromogenic method using calcein/Mn^2+^ complex and the real-time turbidity monitoring to assess the reaction. Then dissemination of L1-producing *S. maltophilia* was investigated from ICU patients in three top hospital in Beijing, China. The results showed that both methods detected the target DNA within 60 min under isothermal conditions (65°C). The detection limit of LAMP was 3.79 pg/μl DNA, and its sensitivity 100-fold greater than that of conventional PCR. All 21 test strains except for *S. maltophilia* were negative for *bla*_L1_, indicative of the high-specificity of the primers for the *bla*_L1_. A total of 22 L1-positive isolates were identified for LAMP-based surveillance of *bla*_L1_ from 105 ICU patients with clinically suspected multi-resistant infections. The sequences of these *bla*_L1_ genes were conservative with only a few sites mutated, and the strains had highly resistant to β-lactam antibiotics. The MLST recovered that 22 strains belonged to seven different *S. maltophilia* sequence types (STs). Furthermore, co-occurrence of *bla*_L1_ and *bla*_L2_ genes were detected in all of isolates. Strikingly, *S. maltophilia* DCPS-01 was recovered to contain *bla*_L1_, *bla*_L2_, and *bla*_NDM-1_ genes, possessing an ability to hydrolyse all β-lactams antibiotics. Our data showed the diversity types of *S. maltophilia* carrying *bla*_L1_ and co-occurrence of many resistant genes in the clinical strains signal an ongoing and fast evolution of *S. maltophilia* resulting from their wide spread in the respiratory infections, and therefore will be difficult to control.

## INTRODUCTION

*Stenotrophomonas maltophilia*, commonly associated with respiratory infections in children and adults, is an emerging Gram-negative MDRO (multi-drug-resistant organism) of global significance (Brooke, 2012). Currently, the incidence of *S. maltophilia* nosocomial infection is increasing, particularly for the immunocompromised (cancer, cystic fibrosis, drug addicts, and AIDS patients), dialysis patients, recipients of organ transplants, in addition to the reported cases of community-acquired *S. maltophilia*. The low outer membrane permeability of *S. maltophilia* renders it inherently resistant to most antibiotics, whilst the production of group 2e β-lactamase (L2) and group 3c β-lactamase (L1) confers resistance to β-lactam containing antibiotics (Alonso and Martínez, 1997). The *bla*_L1_ existed on a 200-kb plasmid, encoding a broad-spectrum metallo-β-lactamase which hydrolyses carbapenems (Walsh et al., 1994; Avison et al., 2001), is usually produced at higher levels (induced) that hydrolyzes almost all known penicillins, cephalosporins, and carbapenems during β-lactam challenge. The ease of acquisition and spread of this antibiotic resistant gene of *bla*_L1_ in *S. maltophilia* emphasizes the need for antibiotic susceptibility testing of clinical isolates. At present, *bla*_L1_ has been identified in some clinical isolates of *S. maltophilia* with important drug resistance against carbapenems (Avison et al., 2001), so detection of *bla*_L1_ plays an important role to indicate the infection of *S. maltophilia* in the clinical work (Alonso and Martínez, 2000; AI-Jasser, 2006; Gould et al., 2006).

Recently, a number of molecular biology techniques have been used to detect different strains of *S. maltophilia* (Nakamura et al., 2010). PCR amplification of the 16S rRNA gene was used to detect *S. maltophilia* in blood samples of patients who are undergoing chemotherapy for acute leukemia or myelodysplastic syndrome (Nakamura et al., 2010). However, PCR requires specialized high-cost instruments and consumables. In addition, *Taq* DNA polymerase in PCR assays can be inactivated by inhibitors present in crude biological samples but the large fragment of *Bst* DNA polymerase (the large fragment of *Bst* DNA polymerase is part of *Bacillus stearothermophilus* DNA polymerase, it has 5′>–3′> DNA polymerase activity) in loop-mediated isothermal amplification (LAMP) assays is more resistant to inhibitors present in crude biological samples (de Franchis et al., 1988; Kaneko et al., 2007). Thus, another rapid, simple and cost effective assay is needed to complement current PCR methods. The LAMP method which was developed in 2000 relies on auto-cycling strand displacement DNA synthesis which proceeds under isothermal conditions, typically within 60 min, and in the presence of *Bst* DNA polymerase (Notomi et al., 2000; Song et al., 2005). In this study, we develop this new method to detect the *bla*_L1_ of *S. maltophilia.* The LAMP method has been shown to amplify target DNA with high-specificity, and it is used widely in the clinical detection of bacteria (Hara-Kudo et al., 2005; Ohtsuka et al., 2005), viruses (Okafuji et al., 2005), parasites (Chen et al., 2011; Kong et al., 2012), and for fetal sex identification (Hirayama et al., 2006).

Data on the prevalence of *bla*_L1_ in *S. maltophilia* from ICU of Chinese hospitals are lacking. The objective of the current study is to develop a rapid, simple assay for *S. maltophilia* and to further investigate the infection status and the species distribution of *bla*_L1_ in clinic. At first, we designed five primer sets which each set targets six or eight sequences on the *bla*_L1_. The specificity and sensitivity of the primers for *bla*_L1_ was confirmed, and the LAMP method used for the detection of *bla*_L1_ in clinical samples. Then, basing on this LAMP assays, dissemination and molecular characterization of L1-producing *S. maltophilia* isolates was investigated at ICU patients in three top hospitals (the hospitals that have large scale and many patients) in Beijing, China.

## MATERIALS AND METHODS

### BACTERIAL ISOLATES, IDENTIFICATION, MLST TYPING, AND ANTIMICROBIAL SUSCEPTIBILITY TESTING

A total of 37 bacterial strains were used in this study to develop the LAMP assays, and their sources are listed in **Table 1**. *S. maltophilia* K279a carrying *bla*_L1_ and *bla*_L2_ with the typical antimicrobial resistance properties was used as the positive control. The other species including common clinical infectious species and homologous species with *S. maltophilia* stored at our laboratory were used for estimating the sensitivity and specificity of the LAMP assay. 105 clinical nasopharyngeal swabs and sputum samples were collected from ICU hospitalized patients with clinically suspected multi-resistant infections in the 307 hospital, 302 hospital, and 301 hospital in China, and species identification was carried out using an automated system (Phoenix and BD systems) and matrix-assisted laser desorption ionization time-of-flight mass spectrometry (MALDI-TOF MS). 16S rDNA and *bla*_L1_ were validated by PCR-based sequencing, and their sequence showed 100% (for 16S rDNA) and 98–100% (for *bla*_L1_) identity with the sequences of previously reported genes, respectively. The allele number for each gene was assigned on the basis of the information in the MLST database ^1^. A combination of the allelic sequences of the seven genes yielded the allelic profile. Antimicrobial susceptibility testing was performed by microbroth dilution according to the Clinical and Laboratory Standards Institute (CLSI, Clinical and Laboratory Standards Institute Performance standards for antimicrobial susceptibility testing; Twentieth informational supplement CLSI Document M100-S20, Wayne, PA, USA 2010.), and Etest strips (bioMérieux) for carbapenems. The carbapenemase activity of isolates was assessed by Etest MBLs.

**Table 1:** Bacterial strains used in the current study.

Species	Source
*Stenotrophomonas maltophilia-*2	Clinical isolate
*S. maltophilia-*17	Clinical isolate
*S. maltophilia*-24	Clinical isolate
*S. maltophilia*-25	Clinical isolate
*S. maltophilia*-36	Clinical isolate
*S. maltophilia*-41	Clinical isolate
*S. maltophilia*-51	Clinical isolate
*S. maltophilia*-58	Clinical isolate
*S. maltophilia*-63	Clinical isolate
*S. maltophilia*-65	Clinical isolate
*S. maltophilia*-66	Clinical isolate
*S. maltophilia*-67	Clinical isolate
*S. maltophilia -*3859	Clinical isolate
*S. maltophilia-*4621	Clinical isolate
*S. maltophilia-*WJ2	Clinical isolate
*S. maltophilia-*K279a	Our microorganism center
*Acinetobacter baumannii* B260	Our microorganism center
*A. baumannii* H18	Our microorganism center
*Bacillus megatherium* 4623	Our microorganism center
Beta hemolytic *Streptococcus* group A CMCC32213	Our microorganism center
*Bordetella pertussis* ATCC 18530	Our microorganism center
*Brucella suis* 3572	Clinical isolate
*Corynebacterium diphtheriae* CMCC38001	Our microorganism center
Enterotoxigenic *Escherichia coli* 44824	Our microorganism center
*Mycobacterium tuberculosis* 8362	Our microorganism center
*Neisseria meningitidis* group B CMCC29022	Our microorganism center
*Salmonella aberdeen* 9264	Our microorganism center
*Salmonella enteritidis* 50326-1	Our microorganism center
*Salmonella paratyphi* 86423	Our microorganism center
*Shigella flexneri* 4536	Our microorganism center
*Shigella sonnei* 2531	Our microorganism center
*Staphylococcus aureus* 2740	Our microorganism center
*Vibrio carchariae* 5732	Our microorganism center
*V. cholera* 3802	Our microorganism center
*V. parahaemolyticus* 5474	Our microorganism center
*Yersinia enterocolitica* 1836	Our microorganism center
*Y. pestis* 2638	Our microorganism center

The strains were screened for the presence of known MBL genes (*bla*_V IM_, *bla*_IMP_, *bla*_SPM-1_, *bla*_GIM-1_, *bla*_SIM-1_, *bla*_AIM-1_, and *bla*_NDM-1_) by PCR with primers as reported previously (Patzer et al., 2009). The strains were also screened for the presence of other β-lactamase genes (*bla*_CTX_, *bla*_CMY_, etc.; Poirel et al., 2007).

### ISOLATION OF GENOMIC DNA

The 37 bacterial strains and the 105 clinical samples were cultured in brain heart infusion (BHI) broth at 37°C according to a standard protocol. Chelex^®^ 100 was used to extract total genomic DNA (including plasmid DNA) from 5 ml overnight bacterial cultures. Briefly, 500 μl bacterial suspension was centrifuged at 10,000 × *g* for 2 min and the supernatant discarded. The pellet was resuspended in 500 μl distilled water and 500 μl Chelex DNA extraction buffer (25 mM NaOH, 10 mM Tris-HCl, 1% Triton X-100, 1% NP-40, 0.1 mM EDTA, 2% Chelex-100) added. The cell suspension was heated in boiling water for 10 min, held on ice for 5 min, and centrifuged at 14,000 × *g* for 2 min. The extracted DNA was used as template in the LAMP and PCR reactions.

As for isolation of DNA from clinical sputum samples, DNA was extracted directly from 200 μl clinical sputum samples with the TIANamp Genomic DNA Kit (TIANGEN Biotech Co., Ltd., Beijing, China). The DNA was purified with the SV GEL and PCR Clean-Up System (Promega Co., USA). The DNA concentration was detected using the Spectrophotometer ND-1000 (Thermo Fisher Scientific, Inc., USA).

### PRIMER DESIGN

A total of 20 *bla*_L1_ in the NCBI GenBank database (GenBank: HQ822273.1; EF126060.1; EF126061.1; AM743169.1; AB294542.1; AJ251814.1; JF705927.1; JF705926.1; EF126051.1; EF126054.1; EF126053.1; EF601224.1; AB294547.1; AB294545.1; AJ291672.1; AF010282.1; AB194306.1; AJ289085.1; AJ289086.1; AB194305.1) were compared, then the sequences of conserved regions were chosen to design the primer sets. Primer Explorer V4 software ^2^ was used to design the outer forward primer (F3), outer backward primer (B3), forward inner primer (FIP), backward inner primer (BIP) and backward loop primer (loop B), used to accelerate the amplification reaction. Mergers of bases are used to circumvent the mutational site (**Table 2**). The FIP and BIP primers were linked by a four thymidine spacer (TTTT). Conventional PCR was performed using primers labeled L1-23-F3 and L1-23-B3. The primers were synthesized by Sangon Biotech Co., Ltd. (Shanghai, China).

**Table 2:** Primers used for the amplification of *bla*_**L1**_.

Primer	Type	Sequence (5’–3’)
L1-23F3	Forward outer	CGGCATGCCACAGATGG
L1-23B3	Backward outer	GCAGCACCGCCGTTTCT
L1-23FIP	Forward inner	TCAATCGCAGGTCCTGCGGTTTTCGGTCACCTGCTGGACAAC
L1-23BIP	Backward inner	CTY(C/T)AGCCATGCGCAY(T/C)GCS(C/G)GATTTTGCATTGGCCGCCACATG
L1-23LB	Loop backward	TCGCCGAGCTCAAGCGT

### LAMP REACTION

A 25 μl reaction volume was used for all LAMP reactions and contained the following components (final concentration): 20 mM Tris-HCl (pH 8.8), 10 mM KCl, 10 mM (NH_4_)_2_SO_4_, 0.1% Tween 20, 0.8 M betaine, 8 mM MgSO_4_, 1.4 mM each dNTP, and 8 U *Bst* DNA polymerase. The amount of primer per reaction was 40 pmol FIP and BIP, 20 pmol LB, 5 pmol F3 and B3. The appropriate amount of DNA template was included in the reaction volume. The LAMP assay proceeded in a reaction tube (Eiken Chemical Co., Ltd., Tochigi, Japan) for 60 min at 65°C.

### DETECTION OF LAMP PRODUCTS

Two independent methods, based on either sample turbidity or fluorescence were used to detect LAMP products. Real-time changes in turbidity were monitored by measuring the optical density (λ_650__nm_) at 6 s intervals, for each LAMP reaction in a Loopamp real-time turbidimeter (LA-320c; Eiken Chemical Co., Ltd.). The changes in turbidity arose from the presence of the amplification by-product Mg_2_P_2_O_7_ (a white precipitate).

The second method used direct visual inspection to assess color changes in the presence of the fluorescent metal ion indicator calcein/Mn^2+^ complex. One microliter of calcein/Mn^2+^ complex (Eiken Chemical Co., Ltd.) was added to 25μl LAMP reaction volume prior to the commencement of the LAMP assay. On completion of the reaction a change in color from orange to green indicated a positive reaction, whilst no color change indicated a negative reaction. The color change was observed by the naked eye under natural light or under UV light at 365 nm.

### PCR DETECTION

A 25 μl reaction volume was used for all PCR reactions and contained the following components: 12.5 μl PCR Taq MasterMix (Tiangen Biotech Co., Ltd.), 9.5 μl double distilled water, 1 μM L1-23F3 and L1-23B3 primers, and DNA template. The oligonucleotide primers used for cloning *bla*_L1_ are F: 5′>-atgcgttctaccctgctcgccttcgcc-3′> and R: 5′>-tcagcgggccccggccgtttccttggccag-3′>. The PCR was carried out as follows: initial PCR activation step, 94°C for 2 min; amplification, 35 cycles of 94°C for 30 s, 59°C for 30 s, and 72°C for 30 s; final extension step, 72°C for 10 min. The amplicons were purified using a PCR Purification Kit (TIANGEN Biotech Co., Ltd., Beijing, China) and sequenced by Beijing AuGCT DNASYN Biotechnology Co., Ltd. The sequences were compared with sequences in the GenBank database.

## RESULTS

### THE OPTIMAL PRIMER SETS FOR LAMP ASSAY

Five primer sets were detected in the same reaction condition using real-time turbidimeter and their turbidity curves were draw at 650 nm according to the amplified results. The optimal primer sets amplified the target sequence with the shortest time among them was chosen for further investigation (see **Table 2**).

### SENSITIVITY OF THE LAMP METHOD FOR *bla*_L1_ DETECTION

The sensitivity of the LAMP method for detecting *bla*_L1_ was evaluated using genomic DNA extracted from *S. maltophilia* K279a (Wizard Genomic DNA purification Kit), serially diluted 10-fold from 379 ng/μl to 0.00379 pg/μl. As shown in **Figure 2A**, the detection limit of the LAMP assay for *bla*_L1_ was 3.790 pg/μl. Visual inspection of the color change, post-LAMP assay, and in the presence of calcein/Mn^2+^ complex confirmed reactions positive (green) and negative (orange) for *bla*_L1_(**Figure 2B**). The results from the two detection methods were in agreement with sensitivity for *bla*_L1_. PCR reactions on the serially diluted DNA using primers L1-23F3 and L1-23B3 were also conducted, and the detection limit for *bla*_L1_ was established as 379 pg/μl (**Figure 2C**).

### SPECIFICITY OF THE LAMP METHOD FOR *bla*_L1_ DETECTION

**FIGURE 1:**
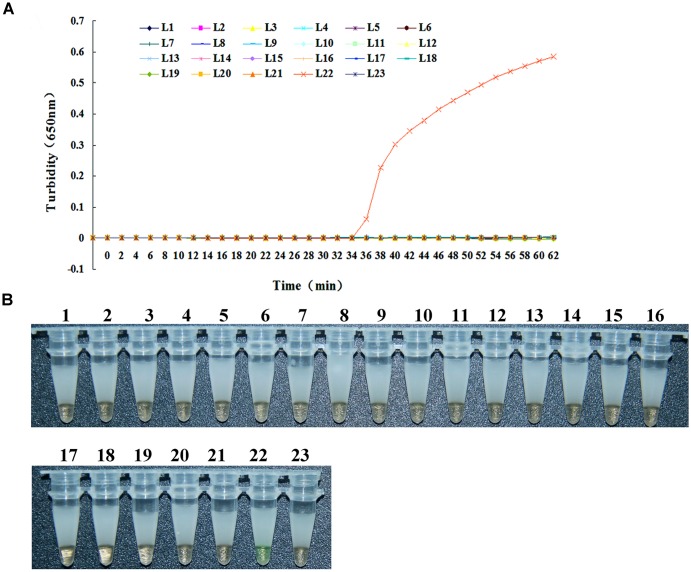
**Specificity of the LAMP method for *bla*_**L1**_ gene detection.** It has two parts, **(A)** is the graphic and **(B)** is the photography of microtubes. The reaction proceeded at 65°C for 65 min. Turbidity was monitored in the Loopamp real-time turbidimeter and the OD_(λ650nm)_ recorded at 6 s intervals. L1, *Brucella suis* 3572; L2, *Bacillus megatherium* 4623; L3, *Vibrio carchariae* 5732; L4, *Acinetobacter baumannii* B260; L5, *Corynebacterium diphtheriae* CMCC38001; L6, *Acinetobacter baumannii* H18; L7, *Mycobacterium tuberculosis* 8362; L8, *Shigella sonnei* 2531; L9, *Shigella flexneri* 4536; L10, *Salmonella enteritidis* 50326-1; L11, *Yersinia enterocolitica* 1836; L12, *Vibrio parahaemolyticus* 5474; L13, *Salmonella paratyphi* 86423; L14, *Neisseria meningitidis group B* CMCC29022; L15, Enterotoxigenic *E. coli* 44824; L16, Beta hemolytic *Streptococcus group A* CMCC32213; L17, *Yersinia pestis* 2638; L18, *Salmonella aberdeen* 9264; L19, *Vibrio cholera* 3802; L20, *Staphylococcus aureus* 2740; L21, *Bordetella pertussis* ATCC 18530*;* L22, positive control (*S. maltophilia -*K279a); L23, negative control (distilled water).

The specificity of the LAMP method for detecting *bla*_L1_ was evaluated using *S. maltophilia* K279a with *bla*_L1_ as the positive control, distilled water as the negative control, and 21 strains without carrying *bla*_L1_ including common clinical infectious species and homologous species with *S. maltophilia* as test subjects. As shown in **Figure 1A**, turbidity increased only when *S. maltophilia* K279a with *bla*_L1_ was used as template DNA in the LAMP assay. When distilled water and the 21 remaining bacterial species were used as template, no changes in turbidity were recorded. These results suggest that the primers had good specificity for *bla*_L1_. In addition, these results are consistent with those obtained using the fluorescent indicator calcein/Mn^2+^ complex. Whereby, only the LAMP assay with *S. maltophilia* K279a with *bla*_L1_ recorded a color change from orange to green, indicative of a positive reaction (**Figure 1B**). Whilst, all test samples negative for *bla*_L1_ and the negative control remained orange, indicative of a negative reaction.

**FIGURE 2:**
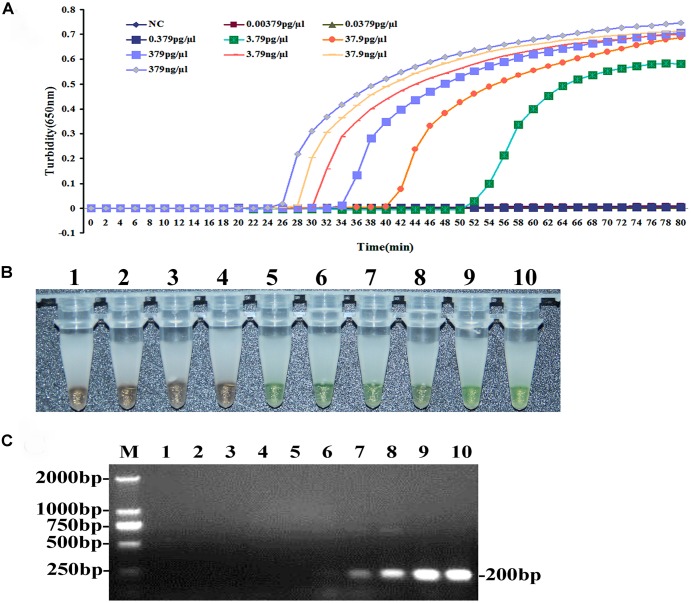
**Comparison of the sensitivities for *bla*_**L1**_ gene detection by LAMP and conventional PCR methods.** Pure genomic DNA extracted from *S. maltophilia-*K279a was diluted tenfold (379.0 ng/μl to 0.00379 pg/μl) and the DNA assayed by LAMP **(A,B)** and PCR **(C)**. **(A)** Turbidity was monitored using the Loopamp real-time turbidimeter and the OD recorded at 650 nm, at 6 s intervals. **(B)** Visual inspection of the color change, post-LAMP assay, and in the presence of calcein/Mn^2+^ complex. **(C)** PCR products were analyzed by 2% agarose gel electrophoresis and stained with ethidium bromide. The DNA marker is D2000 DNA Marker (Tiangen Biotech Co., Ltd.) The size is about 179 bp.

### DISSEMINATION OF L1-PRODUCING *S. maltophilia* IN CLINICAL

A total of 105 clinical sputum samples and nasopharyngeal swabs were collected for LAMP-based surveillance of *bla*_L1_ from 105 ICU patients with clinically suspected multi-resistant infections from the department of Respiratory Diseases in three top hospitals. Ten pairs of sputum samples and nasopharyngeal swabs from healthy people were collected as controls.

**FIGURE 3:**
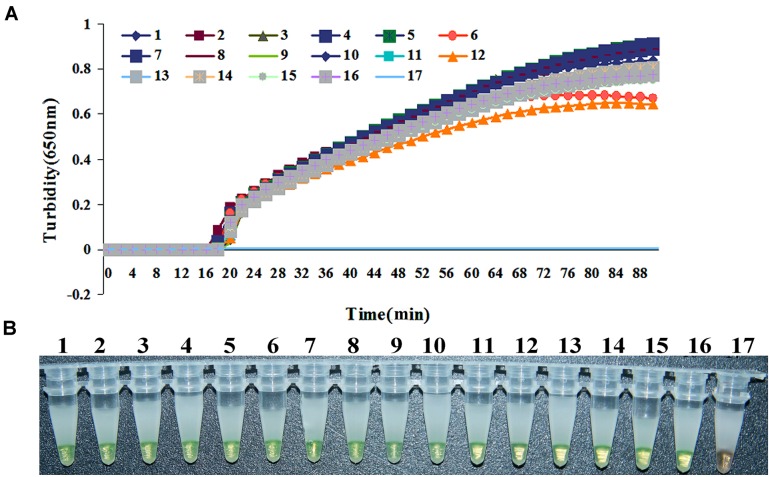
**Loop-mediated isothermal amplification results for 15 *S. maltophilia* strains positive for *bla*_**L1**_ isolated from 15 clinical samples. (A)** Turbidity was monitored using Loopamp, and the OD measured at 650 nm every 6 s. **(B)** Visual inspection of calcein/Mn^2+^ complex associated color changes post-LAMP assay. 1, *S. maltophilia*-2; 2, *S. maltophilia*-17; 3, *S. maltophilia*-24; 4, *S. maltophilia*-25; 5, *S. maltophilia*-36; 6, *S. maltophilia*-41; 7, *S. maltophilia*-51; 8, *S. maltophilia*-58; 9, *S. maltophilia*-63; 10, *S. maltophilia*-65; 11, *S. maltophilia*-66; 12, *S. maltophilia*-67; 13, *S. maltophilia*-3859; 14, *S. maltophilia*-4621; 15, *S. maltophilia* -WJ2; 16, positive control (*S. maltophilia-*K279a); 17, negative control (distilled water).

All clinical samples were analyzed by LAMP and PCR simultaneously. Of the 105 patients samples, 22 were confirmed to be infected with *S. maltophilia* with *bla*_L1_ and 83 negative samples by the LAMP assay (**Figure 3**), whilst the PCR assay detected 13 positive samples, and 92 negative samples. Then 22 strains of *S. maltophilia* were isolated and identified from all of clinical sputum samples and swabs samples, which the positive samples were in accordence with those in LAMP assay. *S. maltophilia* with *bla*_L1_ was positively identified 100% by LAMP and 86.7% by PCR, respectively. None of the samples from healthy people was tested as positive for *bla*_L1_. Thus, the results showed the LAMP assays is more sensitive and the specific than PCR for diagnosis of *S. maltophilia* in clinical practice.

The sequence analysis of the *bla*_L1_ genes from *S. maltophilia* isolates confirmed conservation with the nucleotide sequences of reported genes or with only a few sites mutated. In the MLST analysis of *S. maltophilia*, the results of seven housekeeper genes recovered that 22 strains belonged to different sequence type (ST) including ST4, ST8, ST 25, ST 28, ST29, or ST31, respectively. To further characterize the 22 strains carrying *bla*_L1_ genes, the susceptibility pattern was detected and clearly showed that all isolates had highly resistant to β-lactam antibiotics. The isolates also tested positive for MBLs in both the imipenem-EDTA double-disk synergy test (DDST) and modified Hodge test (MHT).

Furthermore, PCR screening of the isolates were performed for the known MBL genes including *bla*_NDM-1_, *bla*_V IM_, *bla*_IMP_, *bla*_SPM-1_, *bla*_GIM-1_, *bla*_SIM-1_, *bla*_AIM-1_, and *bla*_L2_ (Patzer et al., 2009). PCR yielded products of 22 isolates with expected sizes for *bla*_L1_, and sequencing of these genes showed 100% identities with previously reported genes. It’s interesting to note that the isolate named as *S. maltophilia* DCPS-01 contained L1 and L2 β-lactamase genes with a novel *bla*_NDM-1_ which has attracted wide attention because of its superior resistance to all β-lactam antibiotics, which presented increased carbapenemase activity to all β-lactams (MIC >128 μg/mL for imipenem and meropenem), aminoglycosides and quinolones, and was only susceptible to tigecycline and colistin.

Therefore, our data showed the diversity genotypic features of *S. maltophilia* carrying *bla*_L1_ indicated wide spread in the respiratory infections. Importantly, the emergence of these powerful co-occurring resistance mechanisms described here provides warning that future therapeutic options may be seriously limited.

## DISCUSSION

*Stenotrophomonas maltophilia* is a widespread environmental bacterium that has become a nosocomial pathogen of increasing importance. It is currently the third most common nosocomial non-fermenting bacteria, behind *Pseudomonas aeruginosa* and *Acinetobacter baumannii*, and is associxated with crude mortality rates ranging from 14 to 69% in patients with bacteraemias (Jang et al., 1992; Victor et al., 1994). Of the 1661 antibiotic resistant strains of *S. maltophilia* recovered from 14 hospitals across several regions of China during 2010, the majority (97.5%) were isolated from patients. Approximately 68.3% of strains were isolated from patients ≥60 years of age, whilst only 4.8% were from patients <18 years of age. Most (83.0%) isolates were recovered from sputum and respiratory tract secretions (The data and information from CHINET: CHINET 2010 surveillance of antibiotic resistance in *S. maltophilia* in China). Within hospitals, the bacterium is most often found in water sources, and can be a contaminant of hospital equipment such as nebulizers and intravenous catheters. From these sources, the organism can infect patients, resulting in a wide spectrum of symptoms dependent upon the site of infection, though, most commonly, *S. maltophilia* causes bacteraemias or respiratory tract infections (Denton and Kerr, 1998). β-Lactam resistance is due to the expression of the β-lactamases L1, which together hydrolyze the full range of β-lactam drugs, with the exception of monobactams (Walsh et al., 1994). It is therefore necessary to detect and monitor antibiotic resistance, persistence and spread of *S. maltophilia* within the community and in health care settings.

Loop-mediated isothermal amplification assays are generally less time and labor intensive compared with traditional methods of pathogen detection, in part because the amplification of the target gene is performed at a constant temperature, and the reaction times are usually less than an hour. To date, a method for detecting *S. maltophilia* based on LAMP assays has not been reported. In the current study, we designed primers specific for the metallo-β-lactamase *bla*_L1_ for use in a LAMP assay to detect *S. maltophilia* in clinical samples. Results from the specificity and sensitivity analyses demonstrated that the LAMP method detected genomic DNA at 3.79 pg/μl, and was specific for the β-lactamase *bla*_L1_. In the specificity and sensitivity detection, we only use the *bla*_L1_ of *S. maltophilia* K279a as the target gene, although many other *S. maltophilia* strains do not have the same sequence of *bla*_L1_, the most conserve regions of *bla*_L1_ were chosed to design the decisive primers. In LAMP reaction, it is not true that all the primers should combine with the target sequence. The decisive primers are the FIP and BIP, if the FIP and BIP can combine with the target sequence, the reaction is certain to occur, well, of course, more novel experiments should be made to ensure this conclusion. Although the LAMP method has complex amplification principle, the assay is rapid, easy to operate, highly sensitive and specific, and proceeds under isothermal conditions. We believe this assay would be suitable for use in inspection and quarantine departments and in health care units to test for *S. maltophilia*, and we anticipate its routine use in hospital testing regimes, particularly for rapid clinical testing.

A drawback of the LAMP method is the relatively high false-positive rates; a consequence of the assay’s high sensitivity (Notomi et al., 2000). Strict spatial separation of reagent preparation from the testing area is necessary to avoid contamination. In the current study, a sealing agent was applied to the reaction tube once the reaction mixture had been prepared, and its presence is useful in preventing contamination.

In conclusion, we designed a detection method based on LAMP for the specific, sensitive, rapid, and effective detection of the metallo-β-lactamase *bla*_L1_ of *S. maltophilia.* We believe this technique would greatly benefit hospitals and health units, and we anticipate that LAMP will become the gold standard for the rapid detection of pathogens in clinical samples. At the same time, this report provides new insights into the mechanisms of drug resistance and warning that future therapeutic options may be seriously limited.

## AUTHOR CONTRIBUTIONS

Jing Yuan helped conceive project and designed experiments. Zhan Yang, Wei Liu, and Qian Cui performed and wrote the manuscript. Huan Li, Xiangna Zhao, Xiao Wei, Xuesong Wang, Wenkai Niu, Changqing Bai, Yan Li, and Liuyu Huang designed and executed experiments. Simo Huang, Derong Dong, and Sijing Lu helped to edit the manuscript.

## Conflict of Interest Statement

The authors declare that the research was conducted in the absence of any commercial or financial relationships that could be construed as a potential conflict of interest.

## Footnotes

1 http://pubmlst.org/smaltophilia/

2 http://primerexplorer.jp/e/
